# The Foreign Journals: Chemistry

**Published:** 1840-10

**Authors:** 


### CHEMISTRY.

### *Mode of discovering Adulteration of Essential Oils with Alcohol.* By M. Borsarelli.

M. Borsarelli introduces the essential oil into a cylindrical tube about one inch in diameter and four in length, closed at one end. The tube is to be about two thirds filled with the oil. He then introduces small pieces of chloride of calcium, very dry and free from dust, closes the open end of the tube with a cork, and places it for four or five minutes in a water-bath heated to 100°, occasionally shaking it. The tube is then allowed to cool gradually, and if the oil contain any considerable quantity of alcohol the chloride is entirely dissolved, forming two distinct layers—the superior is the essential oil, the inferior the alcoholic solution of chloride of calcium. If the oil contain but a very small proportion of alcohol the calcareous chloride effloresces, loses its form, and becomes a white mass which adheres to the bottom of the tube. Finally, if the oil contain no alcohol, the chloride not only does not dissolve but retains its proper form.

The same process may be employed to discover the proportion of alcohol mixed with æther, remembering, however, to use a longer tube and not to cork it too tightly.

*Bulletin Général de Thérapeutique.* 30 *Juin*, 1840.

